# Supplementary material for: Insulin-Like Growth Factor-1 Influences Prostate Cancer Cell Growth and Invasion through an Integrin α3, α5, αV, and β1 Dependent Mechanism
Source: Cancers (Basel). 2022 Jan 12;14(2):363. doi: 10.3390/cancers14020363 (PMC8774212; doi:10.3390/cancers14020363)
Supplement: Supplementary file 1 [file cancers-14-00363-s001.zip › cancers-1543656-supplementary.pdf]

Supplementary Material: Insulin-Like Growth Factor-1 Influences Prostate Cancer Cell Growth and Invasion through an Integrin α3, α5, αV, and β1 Dependent Mechanism

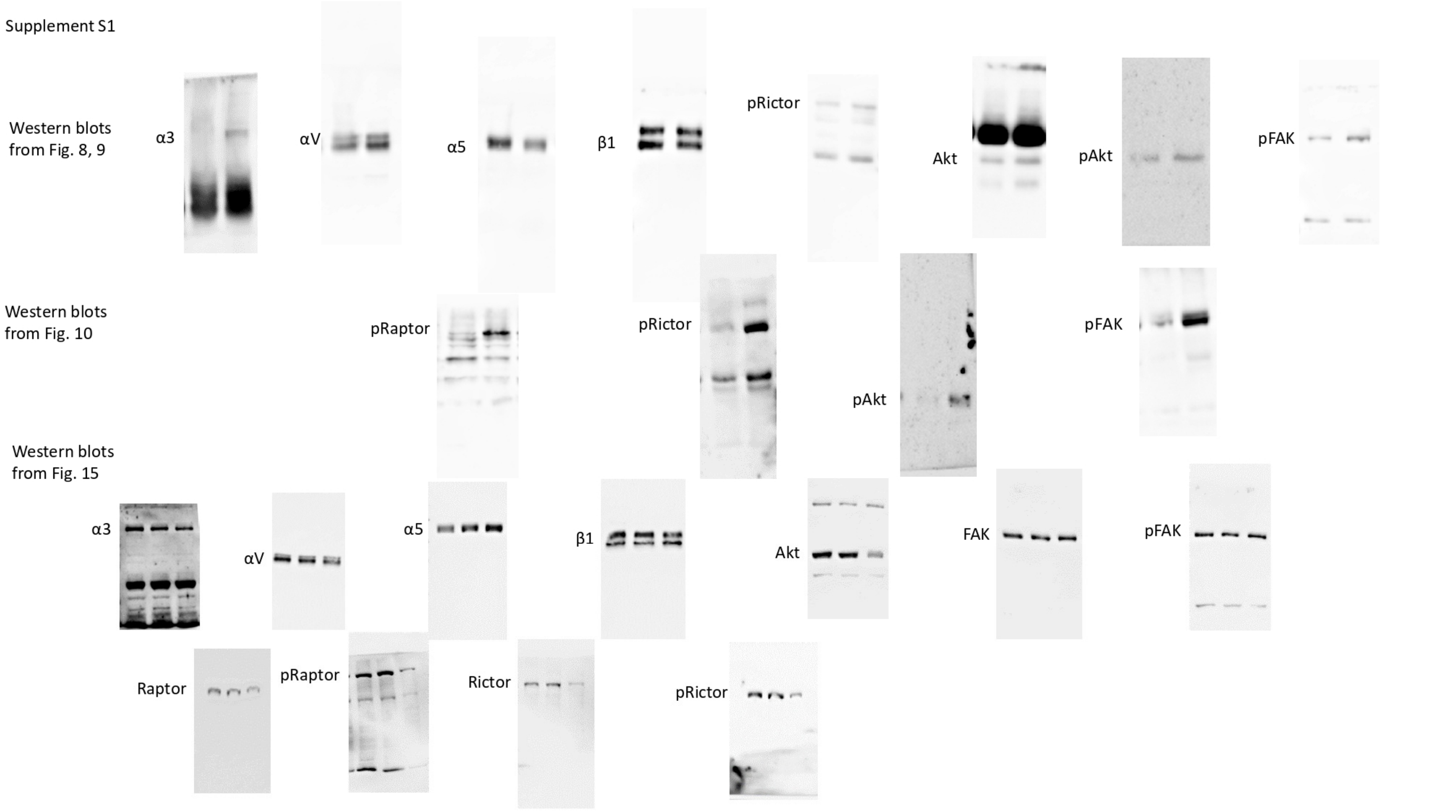

Figure S1. Original Western Blot.
